# Liver iron levels are associated with *HFE*-hemochromatosis genotype, diet, adiposity, and disease in the UK Biobank

**DOI:** 10.1097/HC9.0000000000000883

**Published:** 2026-01-28

**Authors:** Mitchell R. Lucas, Luke C. Pilling, João Delgado, Daniel S. Williamson, Jeremy D. Shearman, Katharine Hutchison, Janice L. Atkins

**Affiliations:** 1 Department of Clinical and Biomedical Sciences, Faculty of Health and Life Sciences, University of Exeter, Exeter, UK; 2 Department of Health and Care Professions, Faculty of Health and Life Sciences, University of Exeter, Exeter, UK; 3 Department of Gastroenterology, South Warwickshire NHS Foundation Trust, UK; 4 Department of Gastroenterology, Royal Devon University Healthcare NHS Foundation Trust, Exeter, UK

**Keywords:** environmental exposure, genetic penetrance, iron overload, magnetic resonance imaging, population studies

## Abstract

**Introduction:**

*HFE* genetic variants, especially C282Y homozygosity (C282Y+/+), can increase systemic iron and cause hemochromatosis, though expression varies. Excess iron can lead to liver disease and liver cancer, yet factors influencing liver iron beyond *HFE* genotype remain unclear. We investigated genetic/environmental factors influencing liver iron, including *HFE* genotype and hemochromatosis diagnosis.

**Methods:**

We analyzed 37,287 European ancestry UK Biobank participants (mean age 64.1, SD: 7.6) with *HFE* genotypes and MRI-estimated liver iron concentrations (MRLIC). Linear regression assessed MRLIC associations with genetic and environmental factors, adjusting for age, sex, and genetic covariates.

**Results:**

Mean MRLIC was highest in undiagnosed C282Y+/+ males and females (2.56 and 2.31 mg/g) versus diagnosed (1.23 and 1.51 mg/g, *p*=0.0001 and 0.0004). Other *HFE* genotypes had nominal increases versus those without *HFE* genetic variants. Higher MRLIC was associated with higher alcohol intake (β=0.11, 95% CI: 0.09–0.11, *p*=6.0×10^−128^; >30 vs. 1–14 units/wk), frequent red/processed meat consumption (β=0.08, 95% CI: 0.07–0.09, *p*=3.7×10^−54^; ≥3 times/week vs. none), high waist-height ratio (β=0.01, 95% CI: 0.006–0.02, *p*=6.4×10^−5^; although magnitude was weak) and genetically predicted transferrin saturation (β=0.22, 95% CI: 0.19–0.26, *p*=3.8×10^−46^). Lower MRLIC was associated with underweight body mass index (β=−0.06, 95% CI: −0.09 to −0.03, *p*=1.1×10^−4^) and proton pump inhibitor use (β=−0.03, 95% CI: −0.04 to −0.03, *p*=3.5×10^−17^).

**Conclusions:**

Undiagnosed C282Y+/+ individuals had excess liver iron versus diagnosed, likely due to treatment. Genetic and environmental factors influence liver iron beyond C282Y+/+. Tailored lifestyle advice could benefit those at risk of hemochromatosis.

## Graphical abstract

**Figure:**
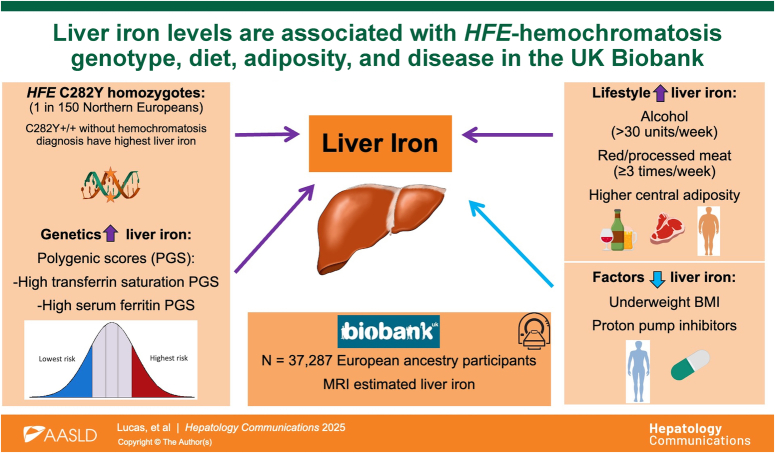

## INTRODUCTION

Hemochromatosis is the most common genetic disorder among people of Northern European descent.^1^ The condition is characterized by iron accumulation, primarily in the liver.^2^ It can be associated with severe clinical outcomes, including liver disease/cancer, joint debilitation, neurodegenerative diseases, and diabetes.^3^^–^^5^ The strongest risk factor is *HFE* C282Y homozygosity (C282Y+/+), with 1 in 150 individuals of Northern European ancestry being C282Y+/+.^6^ While C282Y+/+ exhibit the highest penetrance among *HFE* genotypes, such as elevated transferrin saturation (TSAT) and serum ferritin (SF),^7^ many remain undiagnosed without clinical expression.^3^ Other *HFE* C282Y and H63D genotypes (such as C282Y-H63D compound heterozygotes) have far lower penetrance.^7^

Genetics and environmental factors influence iron status within the body.^8^ Alcohol consumption exacerbates iron accumulation, particularly in C282Y+/+.^9^ Central adiposity has been correlated with lower levels of liver iron,^10^ though findings are mixed.^11^ However, associations between genetic and other modifiers such as vitamin/mineral supplement intake, dietary habits, lifestyle, and other factors have yet to be explored using direct imaging measures of liver iron in large, community-based populations. While previous studies, including our own,^12^ have examined how adiposity affects the risk of clinical outcomes in individuals with *HFE* genotypes, the present study extends this work by investigating MRI-derived liver iron levels as a subclinical biomarker. Understanding how genetics and lifestyle influence hemochromatosis-related phenotypes may help to explain the varied clinical penetrance observed in *HFE* genotypes.

To address these research gaps, we analyzed UK Biobank data, the largest available cohort of *HFE* genotyped participants with MRI estimated liver iron concentrations (MRLIC), to investigate the genetic and environmental factors influencing liver iron status. MRI is a well-established and highly sensitive method for detecting iron in organs, particularly the liver, making it an effective tool for measuring liver iron levels.^13^ Given the low levels of iron accumulated within reticuloendothelial cells in hemochromatosis, it has been inferred that a liver-to-spleen iron ratio (LSIR) may increase the diagnostic specificity of MRI.^14^ Using MRI-derived tissue iron data (liver and spleen) from the UK Biobank, we aim to identify the strongest predictors of iron deposition. First, we compared MRLIC and LSIR by *HFE* genotype, undiagnosed with hemochromatosis, to assess penetrance across each genotype group. Second, we examined genetic and environmental factors associated with iron loading. Finally, we compared mean MRLIC and LSIR across *HFE* genotypes diagnosed with hemochromatosis.

## METHODS

### Study population

The UK Biobank includes over 500,000 community volunteers aged 37–73 years at baseline (2006–2010) from 22 assessment centers across England, Scotland, and Wales. Among this cohort, 451,270 participants were genetically similar to the 1000 Genomes project European reference population,^15^ with *HFE* C282Y (rs1800562) and *HFE* H63D (rs1799945) genotypes from whole exome sequencing (methods by Regeneron).^16^ The UK Biobank has approval from the North West Multi-Centre Research Ethics Committee, and all participants gave written informed consent before the study (Research Ethics Committee reference 11/NW/0382). All research was conducted in accordance with the Declaration of Helsinki. Access to the UK Biobank was granted under application 14631. Participants were informed of baseline health-related findings, but consent did not cover notification of incidental findings from subsequent analyses, including genotyping. To protect participant privacy, the UK Biobank requires that data or aggregate statistics corresponding to fewer than 5 participants cannot be published, nor can data or statistics be reported that allow a participant count of <5 to be derived.^17^

### Baseline assessment (2006–2010)

Socioeconomic status was recorded at baseline using the Townsend Deprivation Index (TDI), which integrates unemployment, lack of car ownership, absence of homeownership, and household overcrowding within a participant’s region of residence.^18^ A greater TDI indicates an increased material disadvantage. Measures of blood glucose were measured using the hemoglobin A1c (HbA1c) test, measured by HPLC analysis on a Bio-Rad VARIANT II Turbo. Total cholesterol and triglycerides were measured using a Beckman Coulter AU5800.

A previously conducted genome-wide association study was performed on 4 key iron-related biomarkers, utilizing data from the Trøndelag Health Study (HUNT), the Michigan Genomics Initiative, and the SardiNIA study.^19^ This research identified 123 genetic loci linked to iron homeostasis and their impact on all-cause mortality.^19^ Genetic variants associated with TSAT and SF from these cohorts (excluding the *HFE* gene) were used to create polygenic risk scores (PGS), which determined participants' genetic likelihood of having high TSAT and SF separately. The PGS were derived from general population genome-wide association studies^19^ and applied here for research purposes; they are not a validated clinical prediction tool.

### Imaging visit (2014–2020)

A subset of 37,287 UK Biobank European ancestry participants (17,780 males; 19,507 females) underwent abdominal MRI between 2014 and 2020; data were available on liver iron (field:40060, n=37,287), spleen iron (field:21170, n=29,411), pancreas iron (field:21091, n=25,907), and liver fat proton density fat fraction (PDFF) (field:40061, n=36,420). MRI liver iron data were also available on 2,859 participants of non-European ancestry: spleen iron n=2,332; pancreas iron n=2,016; liver PDFF n=2,060. Liver iron concentration was quantified using a multiecho spoiled gradient echo (mGRE) sequence acquired on a Siemens 1.5T MAGNETOM Aera scanner, as part of the UK Biobank imaging protocol. T2* decay values were estimated from the multiecho images and converted to liver iron concentration using a validated calibration.^13^^,^^20^ This MRI approach has been previously described and validated against histological and clinical measures.^13^ Clinical thresholds for MRI-derived liver iron concentration were defined as: normal (<1.8 mg/g dry weight; <32 μmol/g), borderline excess (≥1.8 to <3.2 mg/g; 32–57 μmol/g), mild overload (≥3.2 to <7.0 mg/g; 57–125 μmol/g), moderate overload (≥7.0 to 15.0 mg/g; 125–269 μmol/g), and severe overload (>15.0 mg/g; >269 μmol/g). These thresholds reflect validated clinical criteria and align with previously published literature.^21^^,^^22^ LSIR was calculated by dividing liver iron (mg/g) by spleen iron (mg/g). A high LSIR often suggests hemochromatosis-related iron overload. In contrast, a low LSIR is typically associated with secondary iron overload conditions, such as those resulting from blood transfusions or iron-loading anemias.^14^^,^^23^

Participants had their weight, height, waist circumference, and hip circumference measured. Questionnaires collected information on doctor-diagnosed conditions (including hemochromatosis, type 1 and 2 diabetes and viral hepatitis), vitamin and mineral supplement intake, medications (including proton pump inhibitors; PPI), dietary intake, smoking status, alcohol intake, physical activity and education (see Supplemental Methods, http://links.lww.com/HC9/C215 for detailed definitions and categorizations of variables used in this analysis).

### Ascertainment of diagnoses

Ascertainment of disease diagnoses was derived from baseline questionnaires plus International Classification of Diseases, 10th Revision (ICD-10) coded hospital inpatient data (National Health Service Hospital Episode Statistics) from 1996 to the time of the MRI scans (2014–2020). Diseases included hemochromatosis (ICD-10 code E83.1), viral hepatitis (B15, B16, B17, B18, B19, K73), diabetes (type 1 and 2 [E10; E11]), liver fibrosis and cirrhosis (K74), and any liver disease (K70, K71, K72, K73, K74, K75, K76, K77). Treatment history, including data on iron removal therapy such as venesection, was not available. Therefore, we used clinical diagnosis of hemochromatosis as a proxy for potential treatment exposure.

### Statistical Analysis

Descriptive statistics were used to summarize the data in 37,287 participants with (n=58) and without hemochromatosis (n=37,229). We used the Kruskal-Wallis test to assess differences in mean MRLIC, LSIR, liver PDFF, and pancreas iron between *HFE* genotype groups. Normality was checked via Q-Q plots and Shapiro-Wilk tests; data remained non-normal despite log transformations. Logistic regression models were used to compare differences in proportions with borderline excess liver iron and mild iron overload between *HFE* genotype groups.

Participants diagnosed with hemochromatosis were excluded from further regression analyses to avoid bias from potentially receiving ongoing iron monitoring and treatment. Linear regression models examined associations between MRLIC and genetic factors (PGS for TSAT and SF) and environmental factors (waist-to-hip ratio, body mass index [BMI], waist-to-height ratio [WHtR], alcohol intake, smoking, TDI, diet, supplements, PPI use, blood biomarkers, and disease diagnoses). Analyses were conducted within European ancestry participants regardless of genotype (n=37,229), participants without C282Y/H63D variants (n=22,388), C282Y+/+ specifically (n=91), and a combined group of other *HFE* genotypes without hemochromatosis (n=14,750, including H63D+/−, H63D+/+, C282Y+/H63D+, and C282Y+/−). Additionally, analyses were performed among non-European ancestry participants (n=2,859). Models were adjusted for age, sex, and the first 10 ancestry principal components (PCs), as provided by UK Biobank. PC1 and PC2 capture broad continental ancestry differences (eg, European, sub-Saharan African, and East Asian), while PCs 3 to 10 reflect finer-scale structure within and across continental groups, including regional variation within Europe and admixture signals.^24^ Incorporating these components as covariates helps to account for both major and subtle population structure, thereby minimizing confounding in genetic association analyses.^25^

Models were additionally adjusted for covariates: assessment center, red/processed meat consumption, waist-to-hip ratio, alcohol intake, PPI use, education, TDI, smoking, hepatitis, type 2 diabetes, tea drinking, and iron and vitamin C supplements. Covariates were chosen a priori based on established literature on iron metabolism and liver health.^14^^,^^26^^,^^27^ Red and processed meat (heme-iron source), alcohol (hepcidin down-regulation), smoking (greater iron absorption and oxidative stress), tea (tannin-related inhibition of nonheme-iron absorption), PPI use (reduced gastric acidity and iron absorption), iron supplements (direct intake), and vitamin C supplements (enhanced nonheme iron absorption) were included as primary lifestyle and medication exposures. Education, TDI, age, sex, and assessment center were included as confounders given their potential influence on health behaviors and liver outcomes. Waist-to-hip ratio and type 2 diabetes were also included, given their established, albeit complex, relationships with iron regulation, adiposity, and liver health.^27^ Hepatitis status was incorporated to account for underlying liver damage.

We performed additional analysis using Heteroskedasticity-Consistent (HC3) robust standard errors to assess the potential impact of heteroskedasticity in linear regression models between main exposures and MRLIC.

Cox proportional hazards models estimated the associations between MRLIC and risk of liver-related incident outcomes (liver disease, liver fibrosis or cirrhosis, and hemochromatosis), excluding prevalent disease. Models were adjusted for age, sex, and 10 PCs, within participants of European ancestry (n=37,229), without C282Y/H63D variants (n=22,388), and C282Y+/+ (n=91). Sensitivity analyses excluded prevalent anemia cases. We also assessed competing risks for incident liver fibrosis/cirrhosis, including mortality, liver cancer, and hepatitis. Cox proportional hazards assumptions were evaluated using scaled Schoenfeld residuals. Bonferroni correction for 27 regression models set significance at *p*<0.002. Each exposure was modeled individually rather than simultaneously to minimize multicollinearity and to estimate independent associations. While this approach is conservative, it provides clear control of type I errors and supports the robustness and reproducibility of findings in large-scale genetic and epidemiological analyses. Analyses were conducted using R v4.4.0 in the UK Biobank Research Analysis Platform.

### Sensitivity analysis

As a sensitivity analysis, we derived a new outcome variable representing the rate of liver iron accumulation by dividing MRI-derived liver iron concentration by participant age (liver iron/age). This continuous variable was used to explore whether the associations identified in the main analysis were consistent when adjusted for age as a proxy for iron accumulation rate over time. Linear regression models were conducted for each environmental and genetic exposure considered in the main analysis, adjusted for sex and 10 genetic PCs.

## RESULTS

### Males

**FIGURE 1:**
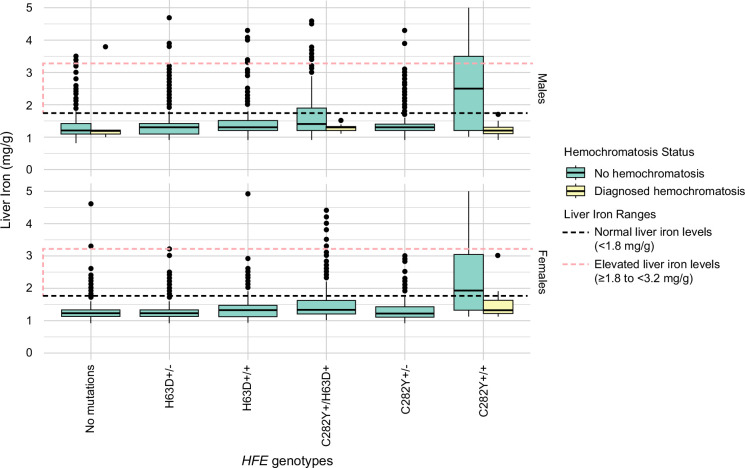
Distribution of MRI liver iron by *HFE* genotype and hemochromatosis diagnosis in the UK Biobank (male n=17,780; female n=19,507) . Abbreviation: mg/g, milligrams per gram.

We analyzed 17,780 males (mean age 64.8 y, SD: 7.7), including 45 C282Y+/+ individuals, of which 22 were diagnosed with hemochromatosis (48.89%). Undiagnosed C282Y+/+ males had the highest mean MRI liver iron concentration (MRLIC) at 2.56 mg/g (SD: 1.4), compared to 1.23 mg/g (SD: 0.2) in those with a hemochromatosis diagnosis (*p*=0.0001). Similarly, MRLIC differed by hemochromatosis status in male C282Y+/H63D+, with values of 1.63 mg/g (SD: 0.6) versus 1.27 mg/g (SD: 0.1) (*p*=0.02). Other *HFE* genotypes had nominal increases in MRLIC compared to those without *HFE* genetic variants (Table 1 shows details on those undiagnosed with hemochromatosis, and Supplemental Table S1, http://links.lww.com/HC9/C215, shows details in those with a diagnosis of hemochromatosis; Figure 1).

**TABLE 1:** Characteristics of UK Biobank participants of European ancestry without a diagnosis of hemochromatosis, by *HFE* C282Y and H63D genotypes and sex

	No C282Y or H63D variants	H63D+/−	H63D+/+	C282Y+/ H63D+	C282Y+/−	C282Y+/+	Total	*p*
Males								
No hemochromatosis diagnosis, n (%)	10,702 (99.95)	4125 (99.95)	362 (97.72)	401 (97.33)	2,125 (99.95)	23 (51.11)	17,738	—
Liver iron (mg/g), mean (SD)	1.27 (0.2)	1.31 (0.3)	1.44 (0.5)	1.63 (0.6)	1.34 (0.3)	2.56 (1.4)	1.30 (0.3)	0.0001
Liver iron groups:								0.0001
Borderline excess liver iron (≥1.8 to <3.2 mg/g), n (%)	325 (3.04)	213 (5.16)	47 (12.98)	109 (27.18)	149 (7.01)	<5	847 (4.78)	—
Mild iron overload (≥3.2 to <7 mg/g), n (%)	<5	7 (0.17)	6 (1.66)	12 (2.99)	<5	10 (43.48)	41 (0.23)	—
Liver-to-spleen ratio, mean (SD)	4.16 (1.0)	4.33 (1.1)	4.72 (1.7)	5.50 (2.2)	4.36 (1.2)	9.26 (4.9)	4.27 (1.1)	0.0001
Liver PDFF (%), mean (SD)	5.52 (5.1)	5.68 (5.2)	5.82 (5.5)	5.88 (5.6)	5.76 (5.4)	5.68 (3.2)	5.6 (5.2)	0.02
Pancreas iron (mg/g), mean (SD)	0.75 (0.1)	0.75 (0.1)	0.75 (0.1)	0.74 (0.1)	0.74 (0.1)	0.74 (0.1)	0.75 (0.1)	0.71
Females								
No hemochromatosis diagnosis, n (%)	11,686 (100)	4,624 (99.96)	443 (99.77)	408 (100)	2262 (99.96)	68 (85.00)	19,491	—
Liver iron (mg/g), mean (SD)	1.22 (0.2)	1.25 (0.2)	1.33 (0.3)	1.47 (0.5)	1.27 (0.2)	2.31 (1.2)	1.25 (0.2)	0.0001
Liver iron groups:								0.0001
Borderline excess liver iron (≥1.8 to <3.2 mg/g), n (%)	188 (1.61)	118 (2.55)	30 (6.77)	63 (15.44)	85 (3.76)	23 (33.82)	507 (2.60)	—
Mild iron overload (≥3.2 to <7 mg/g), n (%)	<5	<5	<5	6 (1.47)	0 (0.00)	16 (23.53)	26 (0.13)	—
Liver-to-spleen ratio, mean (SD)	4.19 (0.8)	4.28 (0.9)	4.53 (1.3)	5.01 (1.9)	4.34 (0.9)	8.17 (4.2)	4.27 (1.0)	0.0001
Liver PDFF (%), mean (SD)	4.22 (4.6)	4.21 (4.5)	4.45 (4.5)	4.36 (4.6)	4.34 (4.7)	4.61 (4.3)	4.24 (4.6)	0.02
Pancreas iron (mg/g), mean (SD)	0.80 (0.1)	0.80 (0.1)	0.81 (0.1)	0.80 (0.1)	0.80 (0.1)	0.80 (0.1)	0.80 (0.1)	0.83

*Note:* Male (n=17,738) and female (n=19,491) genetically similar to the 1000 Genomes Project European Ancestry superpopulation (EUR-like)^15^ with available *HFE* genotypic data and MRI liver iron imaging data in the UK Biobank. Numbers are presented as mean (SD) for continuous variables and n (%) for categorical variables.

Abbreviations: mg/g, milligrams per gram; PDFF, proton density fat fraction.

Undiagnosed C282Y+/+ also had the highest prevalence of mild iron overload (≥3.2 to <7 mg/g) (n=10, 43.48%). Borderline levels of excess liver iron (≥1.8 to <3.2 mg/g) were most common in undiagnosed male C282Y+/H63D+ (n=109, 27.18%), compared to 3.04% in males without C282Y and H63D variants. Undiagnosed C282Y+/+ males had the highest mean LSIR at 9.26 (SD: 4.9), followed by C282Y+/H63D+ males at 5.50 (SD: 2.2), compared to those without *HFE* variants (4.16, SD: 1.0). Other *HFE* genotypes showed a comparable LSIR distribution to males without *HFE* variants undiagnosed with hemochromatosis. There was no significant difference in liver PDFF % between C282Y+/+ individuals without and with hemochromatosis, with values of 5.68% and 6.32%, respectively (*p*=0.69); similar results were observed in C282Y+/H63D+ individuals (5.88% vs. 10.53%, *p*=0.15). Pancreas iron levels did not differ significantly in males between *HFE* genotypes (*p*=0.71 for those without a diagnosis; *p*=0.14 for those with a diagnosis) (Table 1 and Supplemental Table S1, http://links.lww.com/HC9/C215).

### Females

We analyzed 19,507 females (mean age 63.5 y, SD: 7.5), including 80 C282Y+/+ individuals, of which 12 were diagnosed with hemochromatosis (15.00%). Mean liver iron concentration was highest in female C282Y+/+ individuals without a hemochromatosis diagnosis at 2.31 mg/g (SD: 1.2), compared to 1.51 mg/g (SD: 0.5) in those with a hemochromatosis diagnosis (*p*=0.0004). Other *HFE* genotypes had nominal increases in MRLIC compared to those without *HFE* genetic variants (Table 1 and Supplemental Table S1, http://links.lww.com/HC9/C215; Figure 1).

The prevalence of mild iron overload (≥3.2 to <7 mg/g) was also highest in undiagnosed female C282Y+/+ (n=16, 23.53%). Borderline levels of excess liver iron (≥1.8 to <3.2 mg/g) were most common in undiagnosed female C282Y+/+ (n=23, 33.82%) and C282Y+/H63D+ (n=63, 15.44%). Undiagnosed C282Y+/+ females had the highest LSIR at 8.17 (SD: 4.2), followed by C282Y+/H63D+ females at 5.01 (SD: 1.9), while those without *HFE* variants had the lowest LSIR at 4.19 (SD: 0.8). LSIR in other undiagnosed female *HFE* genotypes did not differ compared to those without *HFE* variants. There was no significant difference in liver PDFF % between C282Y+/+ individuals without and with hemochromatosis, with values of 4.61% and 7.34%, respectively (*p*=0.34). Pancreas iron did not differ significantly in females between *HFE* genotype groups, for those undiagnosed (*p*=0.83) and diagnosed (*p*=0.33) (Table 1 and Supplemental Table S1, http://links.lww.com/HC9/C215).

### Associations between genetic and environmental factors with MRLIC in participants undiagnosed with hemochromatosis

**FIGURE 2:**
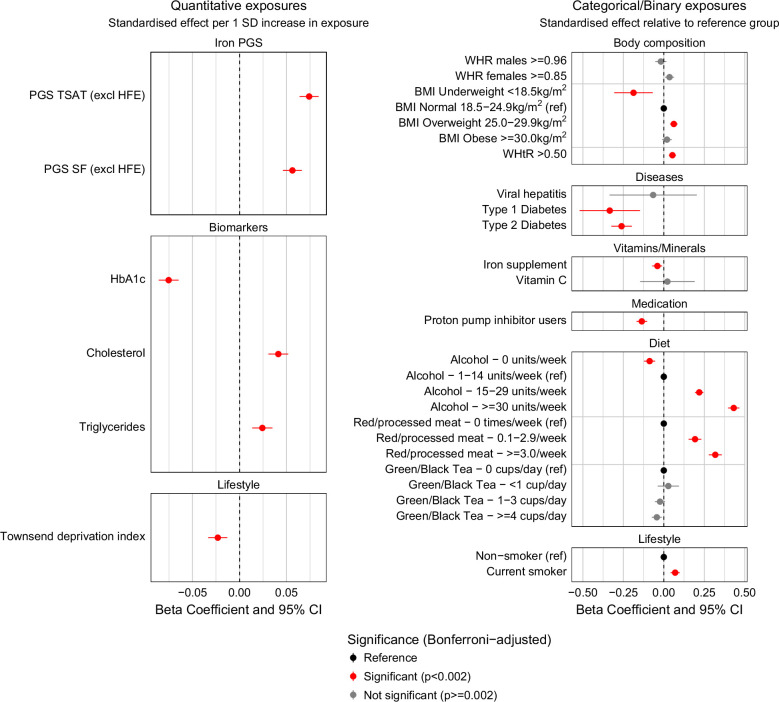
Associations between environmental, clinical, and genetic variables and magnetic resonance liver iron concentration (MRLIC) in UK Biobank participants of European ancestry without a clinical diagnosis of hemochromatosis. Forest plot of standardized beta coefficients with 95% CIs for MRLIC in 37,229 European-ancestry participants without a clinical diagnosis of haemochromatosis. Analyses were adjusted for age, sex, and the first 10 genetic principal components. Quantitative exposures (polygenic scores for transferrin saturation [TSAT] and serum ferritin [SF], biomarkers, and deprivation index) are expressed as standardized effects per 1 SD increase in the exposure. Categorical and binary exposures (body composition, comorbid diseases, vitamin and mineral supplementation, medication use, dietary factors, and lifestyle behaviors) are expressed relative to reference groups: normal BMI, 1−14 units/week alcohol intake, 0 times/week red or processed meat consumption, 0 cups/day tea intake, nonsmoker, no proton pump inhibitor use, no iron or vitamin C supplementation, and absence of type 1 diabetes, type 2 diabetes, or viral hepatitis. *p*-values are Bonferroni-adjusted with a significance threshold of <0.002.

Within 37,229 male and female participants of European ancestry undiagnosed with hemochromatosis, PGS for TSAT and SF were positively associated with MRLIC (β=0.22, 95% CI: 0.19–0.26, *p*=3.8×10^-46^ and β=0.18, 95% CI: 0.15–0.21, *p*=2.1×10^-27^, respectively). Alcohol intake showed a positive dose-response relationship with MRLIC, with consumption of more than 30 units per week showing the strongest association (β=0.11, 95% CI: 0.10–0.12, *p*=6.0×10^-128^) compared to those drinking 1–14 units per week. Red/processed meat consumption exhibited a positive dose-response relationship, with intake of 3 or more times per week most strongly associated with MRLIC (β=0.08, 95% CI: 0.07–0.09, *p*=3.7×10^-54^), compared to those consuming no portions per week. Being a current smoker was also positively associated with MRLIC (β=0.03, 95% CI: 0.02–0.04, *p*=5.1×10^-5^), as was a high WHtR (β=0.01, 95% CI: 0.006–0.02, *p*=6.4×10^-5^); a small magnitude of this effect despite statistical significance. See Supplemental Table S2 and S3, http://links.lww.com/HC9/C215; effect estimates for genetic, clinical, and lifestyle exposures are illustrated as standardized beta coefficients with 95% CIs in Figure 2.

In contrast, being diagnosed with type 1 or 2 diabetes was negatively associated with MRLIC (β=−0.08, 95% CI: −0.12 to −0.03, *p*=9.9×10^-4^ and β=−0.07, 95% CI: −0.08 to −0.05, *p*=8.2×10^-17^, respectively). Similarly, having an underweight BMI was associated with lower MRLIC (β=−0.06, 95% CI: −0.09 to −0.03, *p*=0.0001) compared to a normal BMI, whereas an overweight BMI was associated with higher MRLIC (β=0.02, 95% CI: 0.02–0.03, *p*=4.3×10^-15^). Additionally, TDI was negatively associated with liver iron concentration (β=−0.002, 95% CI: −0.003 to −0.001, *p*=7.8×10^-6^; very small absolute effect size; Figure 2).

PPI medication use was also negatively associated with liver iron (β=−0.03, 95% CI: −0.04 to −0.03, *p*=3.5×10^-17^). Tea drinking was negatively associated with MRLIC among those who consumed 4 or more cups daily (β=−0.01, 95% CI: −0.02 to −0.003, *p*=0.005; magnitude small and nonsignificant after correction) compared to non-tea drinkers; however, this association became nonsignificant after correction for multiple testing (Supplemental Tables S2 and S3, http://links.lww.com/HC9/C215; Figure 2).

After controlling for multiple testing, individuals with *HFE* variants (excluding C282Y+/+) (n=14,750) showed consistently stronger associations with MRLIC compared to those without C282Y or H63D variants (n=22,388). For example, effect estimates were larger in the *HFE* variant (excluding C282Y+/+) group for TSAT PGS (β=0.33 vs. 0.18), hemoglobin A1c (β=−0.004 vs. −0.002), triglycerides (β=0.01 vs. 0.003), type 2 diabetes (β=−0.08 vs. −0.05), PPI use (β=−0.05 vs. −0.02), current smoking (β=0.06 vs. 0.008), high alcohol intake (≥30 units/week: β=0.13 vs. 0.09), and frequent red/processed meat consumption (≥3 times/week: β=0.11 vs. 0.06) (Supplemental Table S3, http://links.lww.com/HC9/C215). Among the 91 undiagnosed C282Y+/+ participants, MRLIC only showed a statistical negative association with tea drinking in those who consumed 1–3 cups daily (β=−0.91, 95% CI: −1.76 to −0.05, *p*=0.04) and 4 or more cups daily (β=−0.99, 95% CI: −1.90 to −0.09, *p*=0.03) compared to non-tea drinkers (Supplemental Table S3, http://links.lww.com/HC9/C215), though neither association remained significant after Bonferroni correction (*p*<0.002).

In non-EUR participants (n=2,859), SF PGS was associated with higher MRLIC (β=0.13, 95%CI: 0.02 to 0.25, *p*=0.03), while the TSAT PGS was not. Comparatively, in the EUR participants, both TSAT and SF PGSs were significantly associated with MRLIC, with larger effect sizes. Alcohol and red/processed meat intake also showed significant positive, dose-response associations with MRLIC in non-EUR, but with slightly stronger effects in EURs. WHtR >0.50 was associated with higher MRLIC in both groups, though the magnitude of this association remained small; other body composition variables (underweight and overweight BMI) were only associated in EURs. Type 2 diabetes was inversely associated with MRLIC in both groups. Type 1 diabetes showed a positive association in non-EURs and a negative one in EURs. PPIs were not significantly associated with MRLIC in non-EURs (Supplemental Table S3, http://links.lww.com/HC9/C215).

In adjusted models, several associations observed in age, sex, and PC-adjusted analyses were no longer statistically significant (Supplemental Table S4, http://links.lww.com/HC9/C215). In contrast, several associations became statistically significant after full adjustment: red/processed meat intake ≥3 times/week was positively associated with liver iron in C282Y+/+ (β=1.13, 95% CI: 0.06–2.21, *p*=0.04), and frequent tea drinking (≥4 cups/day) was associated with lower liver iron in individuals without *HFE* variants (β=–0.009, 95%CI: –0.02 to –0.0008, *p*=0.03). However, these associations were weak in magnitude and did not remain significant after Bonferroni correction.

To investigate the effects of heteroskedasticity, we repeated the main analysis of exposures on liver iron using linear regressions with robust standard errors. Results were consistent, indicating that conclusions were not affected (Supplemental Table S5, http://links.lww.com/HC9/C215).

### Associations between MRLIC and risk of incident disease

Among individuals of European descent, undiagnosed with hemochromatosis at the time of imaging, MRLIC was significantly associated with an increased risk of hemochromatosis (HR=5.40, 95% CI: 3.50–8.33, *p*=2.6×10^−14^). The association between MRLIC and incident diagnosis of liver fibrosis or cirrhosis was not significant after adjusting for multiple statistical testing (HR=0.10, 95% CI: 0.01–0.73, *p*=0.02); Supplemental Table S6, http://links.lww.com/HC9/C215). Proportional hazards assumptions were tested using scaled Schoenfeld residuals. No global violations were observed for the hemochromatosis or liver disease models. For the liver fibrosis/cirrhosis models, a global violation was observed, likely driven by nonproportional effects of PCs and the limited number of events in this analysis; Supplemental Table S7, http://links.lww.com/HC9/C215. In competing risk models, with mortality, liver cancer, or hepatitis prior to the outcome, the association with liver fibrosis/cirrhosis remained unchanged.

### Sensitivity analysis

In sensitivity analyses using liver iron concentration divided by age as the outcome, the direction and strength of associations with most exposures were consistent with the main models; higher alcohol consumption, red/processed meat intake, and higher waist-to-height ratio were positively associated with liver iron/age, while PPI use and underweight BMI were inversely associated. Full regression results are provided in the Supplemental Table S8, http://links.lww.com/HC9/C215.

## DISCUSSION

Hemochromatosis penetrance and clinical severity among *HFE* genotypes vary significantly. C282Y+/+ individuals typically express hemochromatosis-related phenotypes, such as raised TSAT and SF, and associated clinical outcomes more commonly than other *HFE* C282Y and H63D genotypes. However, there is no consensus on whether other *HFE* C282Y and H63D genotypes should be considered risk factors for hemochromatosis-related outcomes caused by iron loading. Our study highlights variations in liver iron by *HFE* genotype and hemochromatosis diagnosis (with the highest levels seen in undiagnosed C282Y+/+) as well as showing associations between genetic and environmental factors and liver iron. Our analysis is based on the largest community-based cohort with available data on *HFE* genotype and MRLIC (n=37,287).

C282Y+/+ undiagnosed with hemochromatosis had the highest liver iron concentrations, with C282Y+/+ diagnosed with hemochromatosis having substantially lower liver iron; likely due to the effectiveness of hemochromatosis diagnosis and treatment in reducing liver iron. Other *HFE* genotypes had nominal increases in MRLIC compared to those without *HFE* genetic variants. We found a strong, significant association between higher MRLIC and an increased risk of hemochromatosis, suggesting that MRLIC is a valuable tool for identifying iron overload. Despite excess liver iron being an established risk factor for liver fibrosis, cirrhosis, and liver cancer,^22^^,^^28^ we found a nominal negative association between MRLIC and risk of liver fibrosis/cirrhosis. However, this association should be treated with caution due to the small sample size (n=39) and did not remain significant after Bonferroni correction.

Alcohol can be a significant co-factor in hemochromatosis, with studies showing that alcohol consumption lowers hepcidin,^29^ and consuming 60 g per day markedly increases the risk of liver fibrosis and cirrhosis.^9^ We demonstrate that alcohol consumption exceeding the recommended weekly limit (1–14 units)^30^ is associated with increased MRLIC in individuals of European ancestry, regardless of genotype, without a diagnosis of hemochromatosis. Importantly, in our study, only a small minority of participants had MRLIC values consistent with mild iron overload and none above this range, meaning that the observed associations with alcohol intake are largely driven by individuals with MRLIC in the normal or elevated but nonclinical iron overload range. Therefore, at a population level, our results show that alcohol intake above the reference range is associated with modest differences in liver iron rather than with clinically significant overload; thus, further research including individuals with iron overload and MRLIC data is required.

Fracanzani et al^31^ studied 452 Italian hemochromatosis patients and found smoking increased the risk of death (HR=2.1, 95% CI: 1.1–3.8; *p*=0.02) and HCC (HR=2.3, 95% CI: 1.2–2.7; *p*=0.01) compared to nonsmokers. We observed an association between being a current smoker (vs. nonsmoker) and higher liver iron in European Ancestry participants of all genotypes without hemochromatosis; however, in our smaller sample of C282Y+/+ (n=91) this association was not significant.

We previously identified that higher central adiposity in male (n=1297) and female (n=1602) C282Y+/+ individuals was associated with increased risks of liver complications.^12^ Pušeljić et al^10^ reported that fat distribution influences liver iron status in hemochromatosis patients (n=52), with intramuscular fat correlating positively (*p*=0.005, *r*_s_=0.382) and visceral subcutaneous fat correlating negatively (*p*<0.001, r_s_=−0.488) with liver iron overload. Powell et al^32^ reported BMI was independently associated with liver fibrosis in both light (OR=2.2, 95% CI: 1.2–4.2; *p*=0.01) and moderate-heavy alcohol drinkers (OR=3.6, 95% CI: 1.2–10.6; *p*=0.02). Our results support the notion that body composition is associated with liver iron status; we observed significant associations between overweight BMI and higher WHtR, with higher liver iron, though the absolute effect size for WHtR was small and should be interpreted with caution.

Iron overload increases the risk of diabetes,^33^ but here we observe a significant negative association between MRLIC and T1D/T2D. The effect a diagnosis of diabetes has on iron is unclear and although lifestyle changes impacting iron status are possible, population-based studies suggest that a diagnosis of type 2 diabetes does not result in drastic health behavior changes.^34^ The liver iron-diabetes association observed here therefore needs further investigation in prospective studies.

European^14^ and American^35^ liver disease guidelines recommend a healthy diet for hemochromatosis patients, avoiding iron and vitamin C supplements and iron-fortified foods, but provide limited advice on red meat. Saliou et al^36^ found that in French C282Y+/+ hemochromatosis patients treated by phlebotomy (n=222), there was a weak association between consuming an iron-rich diet and greater iron overload. Rossi et al^37^ reported that higher consumption of red meat was associated with higher SF in males and females. Similarly, we observe a significant positive association between red/processed meat intake and MRLIC. However, as with alcohol intake, very few participants in our study had MRLIC levels consistent with mild iron overload. The effect estimates are therefore primarily driven by variation within normal and elevated MRLIC ranges, rather than by individuals with clinical iron overload. This distinction is important when interpreting our findings.

Previously, a small clinical trial of 18 C282Y+/+ showed that black tea drinking with meals reduced intestinal iron absorption by around 70%,^38^ while another study showed no significant reduction in iron absorption from a single meal compared with water.^39^ Our results found a negative association between tea consumption and MRLIC in C282Y+/+ individuals without hemochromatosis in models adjusting for age, sex and PCs; this association did not remain significant after Bonferroni correction. While we observed negative associations between tea consumption and liver iron in some analyses, these were of small magnitude and did not remain significant after Bonferroni correction, highlighting that these results should be interpreted with caution.

Over 25% of PPI users adhere to the medication for over a year^40^; extended use can inhibit both nonheme and heme absorption.^41^ Among C282Y+/+ hemochromatosis patients assigned PPIs in a double-blind randomized controlled trial, the need for phlebotomies was significantly decreased versus non-PPI users.^42^ Our findings support this literature, finding that PPI use was associated with lower liver iron within European ancestry participants regardless of genotype. Long-term PPI use can increase the risk of bone fractures^43^ and dementia.^44^ Therefore, despite potential benefits, current evidence does not support routine PPI use for hemochromatosis, and further research is needed to assess safety and efficacy.

Our findings highlight LSIR’s potential in diagnosing hemochromatosis in C282Y+/+ individuals, though we lacked data to track secondary iron overload patterns. C282Y+/+ males undiagnosed with hemochromatosis had the highest LSIR compared to other genotypes, consistent with iron loading within hepatocytes in hemochromatosis. In contrast, other genotypes showed balanced LSIR without signs of iron overload. While spleen iron measurements are less commonly used clinically than liver iron, a study by Sorokin et al^45^ showed their feasibility and relevance in iron metabolism, linking spleen iron to genetic variations in erythrocyte and macrophage-related genes.

While UK Biobank does not include liver biopsy data, prior validation studies have demonstrated that MRI-derived liver iron concentration correlates strongly with histological iron grades, particularly in iron overload conditions.^20^ This supports the reliability of our imaging-based estimates as noninvasive proxies for tissue iron burden.^13^

### Strengths and limitations

We studied the largest available community-based cohort with *HFE* genotypes and MRI data. Measures of SF and TSAT were unavailable in the UK Biobank, but liver MRI is an established, noninvasive method for quantifying tissue iron, comparable to liver biopsy. UK Biobank participants were healthier than the general population at baseline.^46^ However, *HFE* allele frequencies were similar to other studies in the UK.^8^ Due to the absence of venesection treatment data, we used hemochromatosis diagnosis as a proxy, assuming diagnosed individuals are more likely to have received treatment, and excluded diagnosed individuals from the main analysis. Our study was cross-sectional in nature so the causality of associations cannot be inferred. The negative association of liver iron and tea consumption should be interpreted cautiously as we were unable to determine if participants drank tea with their meals or at other times, and tea drinking included both black and green tea.^47^ Another limitation is the modest representation of iron overload in the UK Biobank, with most participants having normal or borderline elevated levels, potentially limiting the study’s ability to investigate the full spectrum of iron loading.

While we applied a PGS for iron traits derived from large-scale genome-wide association study,^19^ this score has not been formally validated as a predictive tool in patient populations. Its use in this study should therefore be considered exploratory and hypothesis-generating rather than clinically applicable at present. Subgroup analyses among C282Y+/+ should be regarded as exploratory, given the limited sample size, wide confidence intervals, and the potential for inflated effect estimates.

Finally, while our findings highlight important subclinical variations in liver iron accumulation, we recognize the ethical considerations surrounding actionable genetic findings such as *HFE* C282Y+/+. Although this variant is included in the American College of Medical Genetics and Genomics Tier 1 list of medically actionable conditions,^48^ the UK Biobank protocol prohibits returning individual genetic results to participants. At enrolment, participants consented not to receive feedback on genetic or clinical findings unless included in specific return-of-results initiatives, which did not encompass *HFE* genotyping. As researchers, we are therefore unable to disclose findings to individuals, even when they may be clinically relevant. This limitation underscores the broader debate around feedback of potentially actionable information in large population cohorts.

### Future studies

Although our study is observational and does not establish causality, our findings have potential implications for clinical diagnosis and management. MRLIC may serve as useful tools for detecting early, subclinical iron accumulation, particularly in genetically susceptible individuals. Furthermore, associations observed between modifiable lifestyle factors such as alcohol intake, dietary habits, adiposity, and smoking and liver iron highlight areas for potential intervention. Future prospective studies should assess whether personalized lifestyle interventions can effectively manage iron accumulation and reduce progression to clinical iron-overload disease, especially among high-risk groups such as C282Y+/+.

Additionally, future research should explore causal relationships between lifestyle and genetic exposures and liver iron accumulation using causal inference approaches, such as Mendelian randomization. Larger-scale imaging studies within C282Y+/+ with deeper clinical phenotyping could clarify variability in iron loading and disease progression. Finally, integrating detailed clinical data, including treatment histories and longitudinal iron indices, could help refine the clinical utility of MRI-derived iron measures and inform optimal management strategies.

## CONCLUSIONS

Male and female C282Y+/+ without a hemochromatosis diagnosis exhibit excess liver iron, whereas other *HFE* genotypes show a low penetrance of iron loading. Liver iron is associated with genetic factors, alcohol consumption, smoking, body composition, PPI medication, and dietary habits. C282Y+/+ diagnosed with hemochromatosis have significantly lower liver iron levels, likely due to treatment effects and lifestyle modifications. Our findings suggest that individuals at risk of hemochromatosis may benefit from tailored advice on lifestyle, diet, and treatment to manage iron accumulation.

## Supplementary Material
