# Supplementary material for: Liver iron levels are associated with HFE-hemochromatosis genotype, diet, adiposity, and disease in the UK Biobank
Source: Hepatol Commun. 2026 Jan 28;10(2):e0883. doi: 10.1097/HC9.0000000000000883 (PMC12854656; doi:10.1097/HC9.0000000000000883)
Supplement: Supplementary file 1 [file hc9-10-e0883-s001.docx]

**Supplementary Material**

Contents

[**Supplementary Methods** 2](#_Toc213140812)

[**eTable 1. Characteristics of male and female UK Biobank participants of European Ancestry, by C282Y and H63D genotypes, with a prevalent diagnosis of hemochromatosis** 3](#_Toc213140813)

[**eTable 2. Characteristics of UK Biobank participants by *HFE* genotype status, without a prevalent diagnosis of hemochromatosis** 4](#_Toc213140814)

[**eTable 3. Associations between environmental and genetic variables and magnetic resonance liver iron concentration in UK Biobank by *HFE* genotype status, without a prevalent diagnosis of hemochromatosis** 6](#_Toc213140815)

[**eTable 4. Adjusted models: Associations between environmental and genetic variables and magnetic resonance liver iron concentration in UK Biobank by *HFE* genotype status, without a prevalent diagnosis of hemochromatosis** 8](#_Toc213140816)

[**eTable 5. Heteroskedasticity-Consistent (HC3) Robust Standard Error Estimates for Exposure Effects in UK Biobank participants of European Ancestry, without a prevalent diagnosis of hemochromatosis** 10](#_Toc213140817)

[**eTable 6. Associations between magnetic resonance liver iron concentration and risk of incident disease outcomes in UK Biobank by *HFE* genotype status, without a prevalent diagnosis of hemochromatosis** 12](#_Toc213140818)

[**eTable 7. Global Schoenfeld residual tests for Cox proportional hazards assumptions in UK Biobank participants of European Ancestry, without a prevalent diagnosis of hemochromatosis** 13](#_Toc213140819)

[**eTable 8.** **Associations between environmental and genetic variables and MRI-derived liver iron accumulation rate (liver iron concentration divided by age) in UK Biobank European ancestry participants, without a prevalent diagnosis of hemochromatosis** 14](#_Toc213140820)

[**References** 16](#_Toc213140821)

##

## **Supplementary Methods**

**Imaging visit (2014-2020) variable definition and categorization**

We calculated body mass index (BMI) as weight (kg) divided by height (m^2^), waist-hip-ratio (WHR) by dividing waist circumference (cm) by hip circumference (cm), and waist-to-height ratio (WHtR) by diving waist circumference (cm) by height (cm). Alcohol intake was categorized by the number of units drank per week groups into ‘0 units per week’, ‘1-14 units per week’ (reference group, based on UK national recommendations)^1^ ’15-29 units per week’ and ‘over 30 units per week’. Using data collected on daily tea drinking, which was derived from being asked “How many cups of tea do you drink each day? (Including black and green tea)”, we categorized data into ‘0 cups’, ‘1-3 cups’ and ‘4 or more cups’. We calculated the number of participants taking iron supplements and vitamin C supplements using data collected from participants being asked "Do you regularly take any of the following?". Vitamin C is of interest due to its relationship with increased iron absorption.^2^ Additionally, we calculated how many participants took proton pump inhibitors (PPI) from participants being asked “In the touch screen you said you are taking regular prescription medications. Can you now tell me what these are?”. Smoking status was defined as ‘current smoker’ based on subjects being asked “Do you smoke tobacco now? and grouped in a binary variable of 0 = ‘No’ and 1 = ‘Yes, on most or all days’ and ‘Only occasionally’. Participants were asked how often they ate processed meat, lamb/mutton, pork, and beef. For each type of meat, participants could choose from the following options: ‘Never’, ‘Less than once a week’, ‘Once a week’, ‘2-4 times a week’, ‘5-6 times a week’, or ‘Once or more daily’. We provided a value for each respective response on meat consumption: (Never=0) (Less than once a week=0.5) (Once a week=1) ( 2-4 times a week=3) (>4 times a week=5.5) then derived a summed total weekly consumption of red /processed meat and recorded as follow ‘0 times/week’ (reference group) and ‘0.1-2.9 times/week’, and ‘≥3.0 times/week’.^3^ Highest educational qualification was ranked as: 0=none, 1=CSEs (Certificate of Secondary Education), 2=GCSEs/O-levels (General Certificate of Secondary Education to age 16), 3=A-levels/NVQ/HND/HNC (further education after age 16), 4=professional qualification, and 5=college/university degree.

## **eTable 1. Characteristics of male and female UK Biobank participants of European Ancestry, by C282Y and H63D genotypes, with a prevalent diagnosis of hemochromatosis**

|  | No C282Y or H63D variants | H63D+/- | H63D+/+ | C282Y+/ H63D+ | C282Y+/- | C282Y+/+ | Total | p-value |
| --- | --- | --- | --- | --- | --- | --- | --- | --- |
|  | **Males** | | | | | | | |
| Hemochromatosis diagnosis, n (%) | 5 (0.05) | <5 | <5 | 11 (2.67) | <5 | 22 (48.89) | 42 | . |
| Liver iron (mg/g), mean (SD) | 1.66 (1.2) | - | - | 1.27 (0.1) | - | 1.23 (0.2) | 1.30 (0.4) | 0.48 |
| Liver iron groups: |  |  |  |  |  |  |  | 0.18 |
| Borderline excess liver iron (≥1.8 to <3.2 mg/g), n (%) | 0 (0.00) | - | - | 0 (0.00) | - | 0 (0.00) | 0 (0.00) | . |
| Mild iron overload (≥3.2 to <7 mg/g), n (%) | <5 | - | - | 0 (0.00) | - | 0 (0.00) | <5 | . |
| Liver-to-spleen ratio, mean (SD) | 7.24 (5.6) | - | - | 4.39 (0.6) | - | 4.18 (1.0) | 4.51 (1.9) | 0.69 |
| Liver PDFF (%), mean (SD) | 10.18 (13.5) | - | - | 10.53 (9.3) | - | 6.32 (6.4) | 7.89 (8.0) | 0.4 |
| Pancreas iron (mg/g), mean (SD) | 0.65 (0.1) | - | - | 0.78 (0.1) | - | 0.75 (0.1) | 0.75 (0.1) | 0.14 |
|  | **Females** | | | | | | | |
| Hemochromatosis diagnosis, n (%) | 0 (0.00) | <5 | <5 | 0 (0.00) | <5 | 12 (15.00) | 16 | . |
| Liver iron (mg/g), mean (SD) | 0 (0.0) | - | - | 0 (0.0) | - | 1.51 (0.5) | 1.46 (0.5) | 0.49 |
| Liver iron groups: |  |  |  |  |  |  |  | 0.86 |
| Borderline excess liver iron (≥1.8 to <3.2 mg/g), n (%) | 0 (0.00) | - | - | 0 (0.00) | - | 0 (0.00) | <5 | . |
| Mild iron overload (≥3.2 to <7 mg/g), n (%) | 0 (0.00) | - | - | 0 (0.00) | - | <5 | <5 | . |
| Liver-to-spleen ratio, mean (SD) | 0 (0.0) | - | - | 0 (0.0) | - | 4.82 (2.5) | 4.75 (2.2) | 0.34 |
| Liver PDFF (%), mean (SD) | 0 (0.0) | - | - | 0 (0.0) | - | 7.34 (9.3) | 7.6 (8.9) | 0.31 |
| Pancreas iron (mg/g), mean (SD) | 0 (0.0) | - | - | 0 (0.0) | - | 0.88 (0.1) | 0.85 (0.1) | 0.33 |

Data are shown for male (n=42) and female (n=16) UK Biobank participants of European ancestry (genetically similar to the 1000 Genomes Project EUR superpopulation) with available *HFE* genotype and MRI-derived liver iron measurements. Participants with a prior diagnosis of hemochromatosis were included. Values are reported as mean (SD) for continuous variables and n (%) for categorical variables. **Abbreviations:** mg/g, milligrams per gram; MRI, magnetic resonance imaging; PDFF, proton density fat fraction; SD, standard deviation. “–” indicates data not shown due to low cell counts (<5).

## **eTable 2. Characteristics of UK Biobank participants by *HFE* genotype status, without a prevalent diagnosis of hemochromatosis**

|  | EUR participants | EUR without *HFE* C282Y and H63D variants | EUR with *HFE* variants excl C282Y homozygotes | EUR C282Y homozygotes | Non-EUR participants |
| --- | --- | --- | --- | --- | --- |
| n | 37,229 | 22,388 | 14,750 | 91 | 2,859 |
| MRI data |  |  |  |  |  |
| Liver iron (mg/g), mean (SD) | 1.27 (0.2) | 1.25 (0.2) | 1.31 (0.3) | 2.37 (1.2) | 1.25 (0.2) |
| Liver PDFF (%), mean (SD) | 4.89 (4.9) | 4.78 (4.8) | 4.82 (4.9) | 4.19 (4.0) | 4.92 (4.8) |
| Pancreas iron (mg/g), mean (SD) | 0.78 (0.1) | 0.78 (0.1) | 0.78 (0.1) | 0.78 (0.1) | 0.77 (0.1) |
| Iron PGS |  |  |  |  |  |
| PGS TSAT (excl *HFE*), mean (SD) | 0.40 (0.1) | 0.32 (0.1) | 0.32 (0.1) | 0.31 (0.1) | 0.40 (0.1) |
| PGS SF (excl *HFE*), mean (SD) | 1.10 (0.1) | 1.10 (0.1) | 1.10 (0.1) | 1.09 (0.1) | 1.08 (0.1) |
| Body composition |  |  |  |  |  |
| WHR ≥0.96 (males), n (%) | 5,668 (32.97) | 3,356 (32.38) | 2,301 (33.98) | 11 (47.83) | 388 (30.12) |
| WHR ≥0.85 (females), n (%) | 5,950 (31.60) | 3,519 (31.16) | 2,413 (32.29) | 18 (27.69) | 487 (33.15) |
| BMI groups (kg/m²), n (%) |  |  |  |  |  |
| Underweight | 270 (0.75) | 158 (0.73) | 109 (0.77) | <5 | 19 (0.69) |
| Normal | 14,488 (40.33) | 8,762 (40.51) | 5,682 (40.00) | 44 (50.00) | 1,091 (39.66) |
| Overweight | 14,862 (41.37) | 8,940 (41.34) | 5,889 (41.46) | 32 (36.36) | 1,118 (40.64) |
| Obese | 6,302 (17.54) | 3,768 (17.42) | 2,525 (17.78) | 9 (10.23) | 523 (19.01) |
| WHtR >0.50, n (%) | 22,364 (64.84) | 13,324 (64.28) | 8,992 (65.76) | 47 (55.29) | 1,726 (65.26) |
| Biomarkers |  |  |  |  |  |
| HbA1c (mmol/mol), mean (SD) | 34.99 (5.1) | 35.16 (5.1) | 34.03 (5.2) | 33.03 (3.8) | 36.14 (6.3) |
| Total cholesterol (mmol/L), mean (SD) | 5.74 (1.1) | 5.75 (1.1) | 5.71 (1.1) | 5.52 (1.0) | 5.65 (1.1) |
| Triglycerides (mmol/L), mean (SD) | 1.64 (1.0) | 1.64 (1.0) | 1.65 (1.0) | 1.51 (0.8) | 1.63 (1.0) |
| Disease status |  |  |  |  |  |
| Viral hepatitis, n (%) | 89 (0.24) | 60 (0.27) | 29 (0.20) | 0 (0.00) | 14 (0.49) |
| Type 1 Diabetes, n (%) | 113 (0.30) | 65 (0.29) | 50 (0.34) | 0 (0.00) | 3 (0.11) |
| Type 2 Diabetes, n (%) | 981 (2.63) | 561 (2.51) | 418 (2.83) | 7 (5.60) | 123 (4.31) |
| Vitamins/Minerals |  |  |  |  |  |
| Iron supplement use, n (%) | 1,174 (3.17) | 743 (3.34) | 428 (2.92) | <5 | 153 (5.41) |
| Vitamin C use, n (%) | 2,690 (7.27) | 1,642 (7.38) | 1,038 (7.08) | 10 (11.24) | 242 (8.55) |
| Medication |  |  |  |  |  |
| Proton pump inhibitors, n (%) | 4,467 (12.00) | 2,670 (11.93) | 1,784 (12.09) | 13 (14.29) | 300 (10.51) |
| Diet |  |  |  |  |  |
| Alcohol intake (units/week), n (%) |  |  |  |  |  |
| 0 units/week | 3,723 (10.15) | 2,242 (10.17) | 1,471 (10.11) | 10 (11.49) | 565 (20.24) |
| 1–14 units/week | 20,521 (55.94) | 12,276 (55.63) | 8,199 (56.37) | 55 (63.22) | 1,614 (57.83) |
| 15–29 units/week | 8,603 (23.45) | 5,243 (23.78) | 3,343 (22.99) | 16 (18.39) | 423 (15.16) |
| ≥30 units/week | 3,837 (10.46) | 2,300 (10.43) | 1,531 (10.53) | 6 (6.90) | 189 (6.77) |
| Red/processed meat consumption (times/week), n (%) |  |  |  |  |  |
| 0 times/week | 2,824 (7.59) | 1,708 (7.63) | 1,099 (7.45) | 17 (18.89) | 321 (11.24) |
| 0.1–2.9 times/week | 16,090 (43.22) | 9,681 (43.24) | 6,366 (43.16) | 42 (46.67) | 1,378 (48.27) |
| ≥3.0 times/week | 18,316 (49.20) | 10,999 (49.13) | 7,285 (49.39) | 32 (35.16) | 1,156 (40.49) |
| Tea intake (cups/day), n (%) |  |  |  |  |  |
| 0 cups/day | 5,340 (14.45) | 3,185 (14.33) | 2,144 (14.64) | 11 (12.36) | 391 (13.83) |
| <1 cup/day | 1,052 (2.85) | 637 (2.87) | 410 (2.80) | 5 (5.62) | 125 (4.42) |
| 1–3 cups/day | 14,532 (39.32) | 8,752 (39.38) | 5,739 (39.18) | 40 (44.94) | 1,316 (46.55) |
| ≥4 cups/day | 16,037 (43.39) | 9,648 (43.42) | 6,356 (43.39) | 33 (37.08) | 995 (35.20) |
| Lifestyle |  |  |  |  |  |
| Current smoker, n (%) | 1,219 (3.30) | 705 (3.17) | 512 (3.49) | <5 | 69 (2.48) |
| Townsend Deprivation Index, mean (SD) | -1.90 (2.7) | 1.88 (2.8) | -1.93 (2.7) | -2.15 (2.5) | -1.03 (3.1) |

Numbers are shown as mean (SD) for continuous variables and n (%) for categorical variables.

**Abbreviations:** BMI, body mass index; excl, excluding; HbA1c, hemoglobin A1c; MRLIC, magnetic resonance liver iron concentration; MRI, magnetic resonance imaging; mmol/L, millimoles per liter; PDFF, proton density fat fraction; PGR, polygenic score; SD, standard deviation; SF, serum ferritin; TSAT, transferrin saturation; WHR, waist-to-hip ratio; WHtR, waist-to-height ratio. PGS for transferrin saturation and serum ferritin were derived from a published GWAS.^4^

## **eTable 3. Associations between environmental and genetic variables and magnetic resonance liver iron concentration in UK Biobank by *HFE* genotype status, without a prevalent diagnosis of hemochromatosis**

|  | **EUR participants** | | **EUR without C282Y and H63D variants** | | **EUR *HFE* variants excl C282Y homozygotes** | | **EUR C282Y homozygotes** | | **Non-EUR participants** | |
| --- | --- | --- | --- | --- | --- | --- | --- | --- | --- | --- |
|  | β (95% CI) | p | β (95% CI) | p | β (95% CI) | p | β (95% CI) | p | β (95% CI) | p |
| **Iron PGS** |  | | | | | | | |  |  |
| PGS TSAT (excl *HFE*) | 0.22 (0.19-0.26) | 3.80E-46 | 0.18 (0.15-0.21) | 9.90E-29 | 0.33 (0.27-0.38) | 1.10E-30 | -2.88 (-6.45-0.69) | 0.11 | 0.11 (-0.006-0.22) | 7.00E-02 |
| PGS SF (excl *HFE*) | 0.18 (0.15-0.21) | 2.10E-27 | 0.17 (0.14-0.20) | 1.60E-25 | 0.20 (0.14-0.25) | 3.80E-11 | 1.03 (-2.68-4.73) | 0.58 | 0.13 (0.02-0.25) | 3.00E-02 |
| **Body composition** |  | | | | | | | | | |
| WHR males ≥0.96 | -0.004 (-0.01-0.004) | 0.33 | -0.008 (-0.02-0.0007) | 0.07 | -0.007 (-0.02-0.009) | 0.38 | 1.08 (-0.79-2.96) | 0.23 | -0.02 (-0.05-0.02) | 0.33 |
| WHR females ≥0.85 | 0.009 (0.002-0.02) | 9.60E-03 | 0.01 (0.004-0.02) | 0.003 | 0.005 (-0.006-0.02) | 0.37 | 0.03 (-0.67-0.73) | 0.93 | 0.02 (-0.008-0.04) | 0.18 |
| BMI |  | | | | | | | | | |
| Underweight | -0.06 (-0.09 - -0.03) | 1.10E-04 | -0.05 (-0.08 - -0.02) | 0.001 | -0.08 (-0.13 - -0.03) | 0.003 | 0.44 (-1.15-2.02) | 0.59 | -0.03 (-0.14-0.08) | 0.57 |
| Normal | Reference | | Reference | | Reference | | Reference | | Reference | |
| Overweight | 0.02 (0.02-0.03) | 4.30E-15 | 0.01 (0.006-0.02) | 6.40E-05 | 0.02 (0.01-0.03) | 4.70E-05 | -0.004 (-0.66-0.66) | 0.99 | 0.02 (-0.002-0.04) | 0.07 |
| Obese | 0.01 (0.003-0.02) | 7.00E-03 | 0.009 (0.002-0.02) | 0.01 | 0.003 (-0.01-0.02) | 0.68 | -0.21 (-1.18-0.76) | 0.67 | 0.01 (-0.01-0.04) | 0.3 |
| WHtR >0.50 | 0.01 (0.006-0.02) | 6.40E-05 | 0.009 (0.003-0.01) | 2.80E-03 | 0.01 (0.003-0.02) | 8.90E-03 | 0.24 (-0.38-0.86) | 0.44 | 0.03 (0.008-0.05) | 7.00E-03 |
| **Biomarkers** |  | | | | | | | | | |
| HbA1c | -0.004 (-0.004 - -0.003) | 4.10E-48 | -0.002 (-0.003 - -0.002) | 3.00E-17 | -0.004 (-0.005 - -0.004) | 1.10E-21 | -0.08 (-0.16-0.001) | 0.05 | -0.002 (-0.003 - - 0.003) | 0.02 |
| Cholesterol | 0.009 (0.007-0.01) | 1.30E-13 | 0.009 (0.007-0.01) | 1.10E-13 | 0.01 (0.007-0.02) | 2.30E-07 | 0.30 (-0.02-0.62) | 0.07 | 0.004 (-0.004-0.01) | 0.35 |
| Triglycerides | 0.006 (0.003-0.009) | 1.20E-05 | 0.003 (-0.0001-0.006) | 0.06 | 0.01 (0.006-0.02) | 8.00E-06 | 0.008 (-0.41-0.43) | 0.97 | 0.03 (-0.007-0.01) | 0.51 |
| **Diseases** |  | | | | | | | | | |
| Viral hepatitis | -0.007 (-0.06-0.04) | 0.78 | 0.03 (-0.02-0.07) | 0.32 | -0.05 (-0.16-0.05) | 0.3 | N/A | N/A | 0.01 (-0.012-0.15) | 0.83 |
| Type 1 Diabetes | -0.08 (-0.12 - -0.03) | 9.90E-04 | -0.07 (-0.12 - -0.02) | 0.003 | -0.09 (-0.17 - -0.008) | 0.31 | N/A | N/A | 0.56 (0.17-0.96) | 0.005 |
| Type 2 Diabetes | -0.07 (-0.08 - -0.05) | 8.20E-17 | -0.05 (-0.07 - -0.04) | 2.10E-10 | -0.08 (-0.11 - -0.05) | 3.90E-09 | -1.24 (-2.62-0.13) | 0.08 | -0.05 (-0.09 - -0.01) | 0.02 |
| **Vitamins/Minerals** |  | | | | | | | | | |
| Iron supplement | -0.03 (-0.05 - -0.02) | 7.60E-06 | -0.02 (-0.04 - -0.008) | 0.002 | -0.04 (-0.07 - -0.02) | 0.01 | -0.67 (-2.16-0.81) | 0.37 | -0.03 (-0.07-0.006) | 0.1 |
| Vitamin C | -0.009 (-0.02-0.0004) | 0.06 | -0.004 (-0.01-0.006) | 0.45 | -0.02 (-0.03-0.003) | 0.09 | -0.62 (-1.46-0.22) | 0.15 | -0.02 (-0.06-0.008) | 0.14 |
| **Medication** |  | | | | | | | | | |
| Proton pump inhibitors | -0.03 (-0.04 - -0.03) | 3.50E-17 | -0.02 (-0.03 - -0.01) | 4.10E-08 | -0.05 (-0.06 - -0.03) | 7.70E-12 | -0.07 (-0.90-0.76) | 0.87 | -0.02 (-0.05-0.01) | 0.28 |
| **Diet** |  | | | | | | | | | |
| Alcohol intake |  | | | | | | | | | |
| 0 units per week | -0.02 (-0.03 - -0.01) | 3.30E-07 | -0.02 (-0.02 - -0.007) | 3.10E-04 | -0.03 (-0.05 - -0.02) | 2.20E-05 | 0.33 (-0.55-1.21) | 0.46 | -0.01 (-0.04-0.008) | 0.22 |
| 1-14 units per week | Reference | | Reference | | Reference | | Reference | | Reference | |
| 15-29 units per week | 0.05 (0.05-0.06) | 7.80E-65 | 0.05 (0.04-0.06) | 1.80E-53 | 0.06 (0.05-0.07) | 2.50E-26 | 0.55 (-0.21-1.31) | 0.15 | 0.04 (0.01-0.07) | 0.007 |
| ≥30 units per week | 0.11 (0.10-0.12) | 6.00E-128 | 0.09 (0.08-0.10) | 7.20E-86 | 0.13 (0.12-0.15) | 1.00E-60 | 1.07 (-0.16-2.30) | 0.09 | 0.09 (0.05-0.13) | 1.70E-05 |
| Red/processed meat consumption |  | | | | | | | | | |
| 0 times/week | Reference | | Reference | | Reference | | Reference | | Reference | |
| 0.1-2.9 times/week | 0.05 (0.04-0.06) | 1.90E-20 | 0.04 (0.03-0.05) | 1.30E-12 | 0.07 (0.05-0.08) | 2.20E-13 | 0.42 (-0.33-1.17) | 0.27 | 0.04 (0.01-0.07) | 4.40E-03 |
| ≥3.0 times/week | 0.08 (0.07-0.09) | 3.70E-54 | 0.06 (0.05-0.07) | 2.20E-35 | 0.11 (0.09-0.12) | 2.60E-31 | 0.56 (-0.29-1.40) | 0.19 | 0.07 (0.04-0.10) | 1.80E-06 |
| Tea intake per day |  | | | | | | | | | |
| 0 cups per day | Reference | | Reference | | Reference | | Reference | | Reference | |
| <1 cup/day | 0.007 (-0.009-0.02) | 0.38 | 0.008 (-0.009-0.02) | 0.37 | 0.003 (-0.03-0.03) | 0.83 | -0.68 (-2.04-0.68) | 0.32 | -0.009 (-0.06-0.04) | 0.71 |
| 1-3 cups/day | -0.006 (-0.01-0.002) | 0.15 | -0.006 (-0.01-0.002) | 0.12 | -0.001 (-00.02-0.01) | 0.86 | -0.91 (-1.76 - -0.05) | 0.04 | 0.004 (-0.02-0.03) | 0.78 |
| ≥4 cups/day | -0.01 (-0.02 - -0.003) | 0.005 | -0.007 (-0.01-0.0007) | 0.07 | -0.01 (-0.03-0.002) | 0.1 | -0.99 (-1.90 - -0.09) | 0.03 | -0.007 (-0.04-0.02) | 0.65 |
| **Lifestyle** |  | | | | | | | | | |
| Current smoker | 0.03 (0.02-0.04) | 5.10E-05 | 0.008 (-0.006-0.02) | 0.26 | 0.06 (0.03-0.08) | 6.80E-06 | 0.21 (-1.65-2.10) | 0.83 | 0.04 (-0.02-0.10) | 0.17 |
| Townsend Deprivation Index | -0.002 (-0.003 - -0.001) | 7.8E-06 | -0.001 (-0.002 - - 0.0005) | 0.002 | -0.003 (-0.005 - - 0.001) | 0.0005 | 0.01 (-0.10 – 0.13) | 0.82 | 0.001 (-0.002-0.004) | 0.49 |

Linear regression beta-coefficients and 95% confidence intervals. Models were adjusted for age, sex, and 10 genetic principal components (PCs) to reduce bias from population stratification.

**Abbreviations:** BMI, body mass index; CI, confidence intervals; excl, excluding; HbA1c, hemoglobin A1c; MRLIC, magnetic resonance liver iron concentration; MRI, magnetic resonance imaging; mmol/L, millimoles per liter; PDFF, proton density fat fraction; PGR, polygenic score; p-value, probability value; SD, standard deviation; SF, serum ferritin; TSAT, transferrin saturation; WHR, waist-to-hip ratio; WHtR, waist-to-height ratio; β, beta-coefficients. PGS for transferrin saturation and serum ferritin were derived from a published GWAS.^4^

## **eTable 4. Adjusted models: Associations between environmental and genetic variables and magnetic resonance liver iron concentration in UK Biobank by *HFE* genotype status, without a prevalent diagnosis of hemochromatosis**

|  | **EUR participants** | | **EUR without C282Y and H63D variants** | | **EUR *HFE* variants excl C282Y homozygotes** | | **EUR C282Y homozygotes** | | **Non-EUR participants** | |
| --- | --- | --- | --- | --- | --- | --- | --- | --- | --- | --- |
|  | β (95% CI) | p | β (95% CI) | p | β (95% CI) | p | β (95% CI) | p | β (95% CI) | p |
| **Iron PGS** |  |  |  |  |  |  |  |  |  |  |
| PGS TSAT (excl *HFE*) | 0.23 (0.20-0.26) | 7.60E-47 | 0.18 (0.15-0.21) | 2.80E-30 | 0.18 (-1.99-2.35) | 0.86 | -3.96 (-8.48-0.55) | 0.08 | 0.11 (-0.003-0.23) | 0.06 |
| PGS SF (excl *HFE*) | 0.18 (0.15-0.21) | 1.90E-27 | 0.16 (0.13-0.20) | 1.90E-23 | 0.20 (0.14-0.26) | 1.40E-11 | 2.87 (-1.30-7.04) | 0.17 | 0.16 (0.04-0.29) | 0.009 |
| **Body composition** |  |  |  |  |  |  |  |  |  |  |
| WHR males ≥0.96 | -0.002 (-0.01-0.007) | 0.71 | -0.005 (-0.01-0.004) | 0.29 | -0.006 (-0.02-0.01) | 0.49 | N/A | N/A | -0.006 (-0.04-0.03) | 0.73 |
| WHR females ≥0.85 | 0.01 (0.006-0.02) | 0.0005 | 0.01 (0.006-0.02) | 0.0002 | 0.009 (-0.003-0.02) | 0.13 | 0.007 (-0.90-0.91) | 0.99 | 0.01 (-0.01-0.04) | 0.34 |
| BMI |  |  |  |  |  |  |  |  |  |  |
| Underweight | -0.03 (-0.06 - -0.003) | 0.03 | -0.04 (-0.07 - -0.007) | 0.02 | -0.06 (-0.11-0.01) | 0.02 | 0.87 (-1.16-2.90) | 0.39 | -0.03 (-0.14-0.08) | 0.61 |
| Normal | Reference | | Reference | | Reference | | Reference | | Reference | |
| Overweight | 0.008 (0.002-0.01) | 0.01 | 0.005 (-0.001-0.01) | 0.12 | 0.01 (0.004-0.03) | 0.006 | 0.02 (-0.95-0.99) | 0.97 | 0.01 (-0.009-0.04) | 0.26 |
| Obese | -0.002 (-0.01-0.005) | 0.49 | 0.003 (-0.005-0.01) | 0.5 | -0.003 (-0.02-0.01) | 0.69 | -0.60 (-2.05-0.84) | 0.41 | 0.009 (-0.02-0.04) | 0.54 |
| WHtR >0.50 | 0.02 (0.009-0.02) | 1.70E-05 | 0.01 (0.005-0.02) | 7.50E-04 | 0.02 (0.01-0.04) | 6.10E-04 | 0.38 (-0.82-1.57) | 0.53 | 0.04 (0.01-0.06) | 4.40E-03 |
| **Biomarkers** |  |  |  |  |  |  |  |  |  |  |
| HbA1c | -0.003 (-0.004 - -0.002) | 1.80E-30 | -0.002 (-0.003 - -0.001) | 8.30E-11 | -0.004 (-0.005 - -0.003) | 4.30E-13 | -0.08 (-0.20-0.04) | 0.17 | -0.002 (-0.003-0.0002) | 0.08 |
| Total cholesterol | 0.005 (0.003-0.007) | 7.10E-05 | 0.006 (0.003-0.008) | 5.40E-06 | 0.006 (0.002-0.01) | 0.007 | -0.008 (-0.43-0.41) | 0.97 | -0.0004 (-0.009-0.009) | 0.93 |
| Triglycerides | 0.009 (0.006-0.01) | 4.40E-09 | 0.004 (0.0007-0.007) | 0.02 | 0.02 (0.01-0.02) | 7.90E-10 | -0.13 (-0.61-0.36) | 0.61 | 0.003 (-0.008-0.01) | 0.62 |
| **Disease status** |  |  |  |  |  |  |  |  |  |  |
| Viral hepatitis | 0.003 (-0.05-0.05) | 0.91 | 0.04 (-0.01-0.09) | 0.13 | -0.04 (-0.14-0.06) | 0.42 | N/A | N/A | 0.01 (-0.13-0.15) | 0.87 |
| Type 1 Diabetes | -0.06 (-0.11 - -0.01) | 0.01 | -0.06 (-0.10 - -0.007) | 0.02 | -0.07 (-0.15-0.01) | 0.09 | N/A | N/A | 0.62 (0.19-1.04) | 0.004 |
| Type 2 Diabetes | -0.06 (-0.08-0.05) | 6.50E-15 | -0.05 (-0.07 - -0.04) | 9.80E-10 | -0.07 (-0.10-0.05) | 1.60E-07 | 1.24 (-2.94-0.46) | 0.15 | -0.05 (-0.10 - -0.006) | 0.03 |
| **Vitamins/Minerals** |  |  |  |  |  |  |  |  |  |  |
| Iron supplement | -0.02 (-0.03 - -0.004) | 0.01 | -0.007 (-0.002-0.007) | 0.31 | -0.03 (-0.06 - -0.001) | 0.04 | 0.51 (-1.20-2.22) | 0.55 | -0.01 (-0.05-0.03) | 0.53 |
| Vitamin C | -0.004 (-0.01-0.006) | 0.48 | -0.0001 (-0.01-0.01) | 0.98 | -0.007 (-0.02-0.01) | 0.45 | -0.86 (-1.92-0.20) | 0.11 | -0.02 (-0.05-0.01) | 0.23 |
| **Medication** |  |  |  |  |  |  |  |  |  |  |
| Proton pump inhibitors | -0.03 (-0.04 - -0.03) | 1.40E-18 | -0.02 (-0.03 - -0.02) | 6.40E-10 | -0.05 (-0.06 - -0.04) | 6.50E-13 | 0.01 (-0.93-0.96) | 0.98 | -0.01 (-0.05-0.02) | 0.37 |
| **Diet** |  |  |  |  |  |  |  |  |  |  |
| Alcohol intake |  |  |  |  |  |  |  |  |  |  |
| 0 units/week | -0.02 (-0.03 - -0.009) | 9.40E-05 | -0.01 (-0.02 - -0.003) | 9.80E-03 | -0.03 (-0.04 - -0.01) | 0.001 | 0.21 (-0.74-1.16) | 0.66 | -0.01 (-0.03-0.01) | 0.38 |
| 1-14 units/week | Reference | | Reference | | Reference | | Reference | | Reference | |
| 15-29 units/week | 0.05 (0.04-0.06) | 4.40E-54 | 0.05 (0.04-0.05) | 2.70E-46 | 0.06 (0.05-0.07) | 4.40E-23 | 0.29 (-0.52-1.10) | 0.48 | 0.04 (0.005-0.07) | 0.02 |
| ≥30 units/week | 0.10 (0.09-0.11) | 5.00E-109 | 0.08 (0.07-0.09) | 1.10E-71 | 0.13 (0.11-0.14) | 9.50E-55 | 1.13 (-0.43-2.68) | 0.15 | 0.09 (0.04-0.13) | 7.90E-05 |
| Red/processed meat consumption |  |  |  |  |  |  |  |  |  |  |
| 0 times/week | Reference | | Reference | | Reference | | Reference | | Reference | |
| 0.1-2.9 times/week | 0.05 (0.04-0.06) | 8.30E-21 | 0.04 (0.03-0.05) | 7.60E-12 | 0.07 (0.05-0.09) | 7.30E-13 | 1.09 (0.05-2.12) | 0.41 | 0.05 (0.02-0.08) | 0.003 |
| ≥3.0 times/week | 0.07 (0.06-0.09) | 2.40E-45 | 0.06 (0.05-0.07) | 4.50E-28 | 0.10 (0.08-0.12) | 7.50E-26 | 1.13 (0.06-2.21) | 0.04 | 0.07 (0.04-0.11) | 1.10E-05 |
| Tea intake per day |  |  |  |  |  |  |  |  |  |  |
| 0 cups/day | Reference | | Reference | | Reference | | Reference | | Reference | |
| <1 cup/day | 0.005 (-0.01-0.02) | 0.54 | 0.006 (-0.01-0.02) | 0.49 | 0.001 (-0.03-0.03) | 0.95 | -1.13 (-2.68-0.41) | 0.15 | -0.007 (-0.06-0.04) | 0.78 |
| 1-3 cups/day | -0.008 (-0.02 - -0.0002) | 0.04 | -0.008 (-0.02-0.0003) | 0.06 | -0.008 (-0.02-0.006) | 0.29 | -1.03 (-2.02 - -0.05) | 0.04 | 0.008 (-0.02-0.04) | 0.57 |
| ≥4 cups/day | -0.01 (-0.02 - -0.005) | 1.20E-03 | -0.009 (-0.02 - -0.0008) | 0.03 | -0.02 (-0.03 - -0.003) | 0.02 | -0.81 (-1.83-0.21) | 0.12 | 0.003 (-0.03-0.03) | 0.85 |
| **Lifestyle** |  |  |  |  |  |  |  |  |  |  |
| Current smoker | 0.01 (-0.0006-0.03) | 0.06 | -0.004 (-0.02-0.01) | 0.59 | 0.04 (0.01-0.06) | 0.004 | 0.72 (-1.52-2.95) | 0.52 | 0.04 (-0.02-0.10) | 0.21 |
| Townsend Deprivation Index | -0.0009 (-0.006-0.004) | 0.71 | 0.0008 (-0.004-0.006) | 0.75 | -0.002 (-0.01-0.008) | 0.76 | N/A | N/A | 0.00005 (-0.02-0.02) | 0.99 |

Linear regression beta-coefficients and 95% confidence intervals. Models were adjusted for age, sex, 10 principal components, assessment centre, red/processed meat consumption, WHR, alcohol intake, PPI use, education, Townsend Deprivation Index, smoking, hepatitis, type 2 diabetes, tea drinking, iron and vitamin C supplement intake.

**Abbreviations:** BMI, body mass index; CI, confidence intervals; HbA1c, hemoglobin A1c; MRLIC, magnetic resonance liver iron concentration; MRI, magnetic resonance imaging; mmol/L, millimoles per litre; PDFF, proton density fat fraction; PGR, polygenic score; p, probability value; SD, standard deviation; SF, serum ferritin; TSAT, transferrin saturation; WHR, waist-to-hip ratio; WHtR, waist-to-height ratio; β, beta-coefficients. PGS for transferrin saturation and serum ferritin were derived from a published GWAS.^4^

## **eTable 5. Heteroskedasticity-Consistent (HC3) Robust Standard Error Estimates for Exposure Effects in UK Biobank participants of European Ancestry, without a prevalent diagnosis of hemochromatosis**

| **Model / Exposure** | **Estimate** | **Robust SE** | **t-value** | **p-value** | **95% CI (Lower)** | **95% CI (Upper)** |
| --- | --- | --- | --- | --- | --- | --- |
| **Iron PGS** |  |  |  |  |  |  |
| PGS TSAT (excl *HFE*) | 0.07 | 0.01 | 14.22 | 8.80E-46 | 0.06 | 0.08 |
| PGS SF (excl *HFE*) | 0.06 | 0.01 | 10.70 | 1.11E-26 | 0.05 | 0.07 |
| **Body composition** |  |  |  |  |  |  |
| WHR males ≥0.96 | 0.00 | 0.00 | -1.02 | 3.06E-01 | -0.01 | 0.00 |
| WHR females ≥0.85 | 0.01 | 0.00 | 2.49 | 1.28E-02 | 0.00 | 0.02 |
| BMI |  |  |  |  |  |  |
| Underweight | 0.02 | 0.00 | 7.90 | 2.94E-15 | 0.02 | 0.03 |
| Normal | Reference | | | | | |
| Overweight | 0.01 | 0.00 | 2.81 | 4.94E-03 | 0.00 | 0.02 |
| Obese | -0.06 | 0.02 | -3.12 | 1.80E-03 | -0.09 | -0.02 |
| WHtR >0.50 | 0.01 | 0.00 | 4.96 | 6.95E-07 | 0.01 | 0.02 |
| **Biomarkers** |  |  |  |  |  |  |
| HbA1c | 0.00 | 0.00 | -13.99 | 2.25E-44 | 0.00 | 0.00 |
| Cholesterol | 0.01 | 0.00 | 7.57 | 3.81E-14 | 0.01 | 0.01 |
| Triglycerides | 0.01 | 0.00 | 3.94 | 8.10E-05 | 0.00 | 0.01 |
| **Diseases** |  |  |  |  |  |  |
| Viral hepatitis | -0.02 | 0.03 | -0.56 | 5.73E-01 | -0.07 | 0.04 |
| Type 1 Diabetes | -0.08 | 0.02 | -5.14 | 2.79E-07 | -0.11 | -0.05 |
| Type 2 Diabetes | -0.06 | 0.01 | -9.88 | 5.41E-23 | -0.08 | -0.05 |
| **Vitamins/Minerals** |  |  |  |  |  |  |
| Iron supplement | 0.01 | 0.02 | 0.25 | 8.01E-01 | -0.04 | 0.05 |
| Vitamin C | -0.01 | 0.01 | -0.89 | 3.73E-01 | -0.03 | 0.01 |
| **Medication** |  |  |  |  |  |  |
| Proton pump inhibitors | -0.03 | 0.00 | -8.62 | 7.08E-18 | -0.04 | -0.03 |
| **Diet** |  |  |  |  |  |  |
| Alcohol intake |  |  |  |  |  |  |
| 0 units per week | -0.02 | 0.00 | -5.83 | 5.54E-09 | -0.03 | -0.02 |
| 1-14 units per week | Reference | | | | | |
| 15-29 units per week | 0.06 | 0.00 | 18.60 | 7.56E-77 | 0.05 | 0.07 |
| ≥30 units per week | 0.12 | 0.01 | 22.39 | 2.96E-110 | 0.11 | 0.13 |
| Red/processed meat consumption |  |  |  |  |  |  |
| 0 times/week | Reference | | | | | |
| 0.1-2.9 times/week | 0.05 | 0.00 | 12.02 | 3.07E-33 | 0.04 | 0.06 |
| ≥3.0 times/week | 0.09 | 0.00 | 21.11 | 2.51E-98 | 0.08 | 0.10 |
| Tea intake per day |  |  |  |  |  |  |
| 0 cups per day | Reference | | | | | |
| <1 cup/day | 0.01 | 0.01 | 1.08 | 2.78E-01 | -0.01 | 0.03 |
| 1-3 cups/day | 0.00 | 0.00 | -1.11 | 2.69E-01 | -0.01 | 0.00 |
| ≥4 cups/day | -0.01 | 0.00 | -2.81 | 4.92E-03 | -0.02 | 0.00 |
| **Lifestyle** |  |  |  |  |  |  |
| Current smoker | 0.03 | 0.01 | 4.42 | 9.86E-06 | 0.02 | 0.05 |
| Townsend Deprivation Index | 0.00 | 0.00 | -4.74 | 2.18E-06 | 0.00 | 0.00 |

HC3 heteroskedasticity-consistent robust standard errors for the association between each exposure and MRI derived liver iron concentration in linear regression models. Estimates (β) represent the change in the outcome per unit increase (continuous exposures) or relative to the reference category (categorical exposures), with corresponding 95% confidence intervals and p-values. Robust standard errors (HC3) were applied to investigate effects of possible heteroskedasticity. **Abbreviations:** BMI, body mass index; excl, excluding; HbA1c, hemoglobin A1c; MRLIC, magnetic resonance liver iron concentration; MRI, magnetic resonance imaging; PGR, polygenic score; SD, standard deviation; SF, serum ferritin; TSAT, transferrin saturation; WHR, waist-to-hip ratio; WHtR, waist-to-height ratio. PGS for transferrin saturation and serum ferritin were derived from a published GWAS.^4^

## **eTable 6. Associations between magnetic resonance liver iron concentration and risk of incident disease outcomes in UK Biobank by *HFE* genotype status, without a prevalent diagnosis of hemochromatosis**

| **Outcome** | **EUR participants** | **EUR without *HFE* C282Y/H63D variants** | **C282Y homozygotes** |
| --- | --- | --- | --- |
| **Incident N** | 37,229 | 22,388 | 91 |
| **Any liver disease**, n (%) | 442 (1.19%) | 230 (1.03%) | <5 |
| **Liver fibrosis/cirrhosis**, n (%) | 39 (0.10%) | 22 (0.10%) | <5 |
| **Hemochromatosis**, n (%) | 15 (0.04%) | 6 (0.03%) | 5 (4.00%) |
| **All-cause mortality**, n (%) | 670 (1.80%) | 390 (1.74%) | <5 |
| **Model 1** |  |  |  |
| Any liver disease (HR [95% CI], p) | 0.84 (0.56–1.26), p=0.40 | 1.12 (0.58–2.16), p=0.74 | N/A |
| Liver fibrosis/cirrhosis (HR [95% CI], p) | 0.10 (0.01–0.73), p=0.02 | 0.11 (0.007–1.87), p=0.13 | N/A |
| Hemochromatosis (HR [95% CI], p) | 5.40 (3.50–8.33), p=2.6×10⁻¹⁴ | 4.67 (0.38–58.11), p=0.23 | 0.79 (0.26–2.40), p=0.68 |
|  |  |  |  |
| **Model 2: Excluding any diagnosis of anaemia** |  |  |  |
| N | 36,466 | 21,914 | 89 |
| Any liver disease (HR [95% CI], p) | 0.92 (0.61–1.39), p=0.70 | 1.34 (0.68–2.64), p=0.40 | N/A |
| Liver fibrosis/cirrhosis (HR [95% CI], p) | 0.11 (0.01–1.02), p=0.052 | 0.09 (0.003–2.80), p=0.17 | N/A |
| Hemochromatosis (HR [95% CI], p) | 5.78 (3.73–8.95), p=3.8×10⁻¹⁵ | 7.49 (0.81–68.95), p=0.08 | 0.68 (0.20–2.31), p=0.54 |
|  |  |  |  |

Cox proportional hazards models were used throughout. Models 1 and 2 – adjusted for age, sex, and 10 PCs.

**Abbreviations**: EUR, European; PCs, principal components; BMI, body mass index; NAFLD, non-alcoholic fatty liver disease; MRI, magnetic resonance imaging. MRLIC, MRI liver iron concentration.

## **eTable 7. Global Schoenfeld residual tests for Cox proportional hazards assumptions in UK Biobank participants of European Ancestry, without a prevalent diagnosis of hemochromatosis**

|  | Liver disease | | Fibrosis/cirrhosis | | Hemochromatosis | | Liver disease  w/o anaemia | | Fibrosis/cirrhosis  w/o anaemia | | Hemochromatosis  w/o anaemia | |
| --- | --- | --- | --- | --- | --- | --- | --- | --- | --- | --- | --- | --- |
| Term | **ChiSq** | **p-value** | **ChiSq** | **p-value** | **ChiSq** | **p-value** | **ChiSq** | **p-value** | **ChiSq** | **p-value** | **ChiSq** | **p-value** |
| GLOBAL | 7.82 | 0.80 | 22.71 | 0.03 | 7.03 | 0.86 | 9.64 | 0.65 | 26.53 | 0.01 | 6.12 | 0.91 |

Results from global scaled Schoenfeld residual tests for the proportional hazards assumption in the primary analyses with EUR sample and repeated excluding anaemia analyses for liver disease, liver fibrosis/cirrhosis, and hemochromatosis. No violations were observed for the liver disease or hemochromatosis models in either cohort (all p > 0.64). Borderline non-proportional hazards were detected for the full fibrosis/cirrhosis model (p = 0.03) and stronger evidence of violation in the anaemia-excluded subset (p = 0.005), likely driven by non-proportional effects of principal components in the model and small samples of liver fibrosis/cirrhosis diagnoses.

## **eTable 8.** **Associations between environmental and genetic variables and MRI-derived liver iron accumulation rate (liver iron concentration divided by age) in UK Biobank European ancestry participants, without a prevalent diagnosis of hemochromatosis**

| Variable | β | Lower 95% CI | Upper 95% CI | p | β (x1000) | Lower 95% CI (x1000) | Upper 95% CI (x1000) |
| --- | --- | --- | --- | --- | --- | --- | --- |
| Iron PGS |  |  |  |  |  |  |  |
| PGS TSAT (excl *HFE*) | 0.07 | 0.06 | 0.08 | 2.34E-36 | 70 | 60 | 80 |
| PGS SF (excl *HFE*) | 0.19 | 0.15 | 0.22 | 4.91E-21 | 190 | 150 | 220 |
| Body composition |  |  |  |  |  |  |  |
| WHR males ≥0.96 | -0.0005 | -0.0007 | -0.0004 | 1.19E-12 | -0.5 | -0.7 | -0.4 |
| WHR females ≥0.85 | -0.0003 | -0.0004 | -0.0002 | 3.00E-06 | -0.3 | -0.4 | -0.2 |
| BMI |  |  |  |  |  |  |  |
| Underweight | -0.0002 | -0.0003 | -0.0001 | 8.00E-05 | -0.2 | -0.3 | -0.1 |
| Normal | Reference | | | | | | |
| Overweight | 0.0001 | -2.00E-05 | 0.0002 | 0.11 | 0.1 | -0.02 | 0.2 |
| Obese | -0.0009 | -0.0001 | -0.0004 | 0.0005 | -0.9 | -0.1 | -0.4 |
| WHtR >0.50 | -0.002 | -0.003 | -0.002 | 5.32E-12 | -2 | -3 | -2 |
| Biomarkers |  |  |  |  |  |  |  |
| HbA1c | -0.0001 | -0.0002 | -0.0001 | 1.17E-220 | -0.1 | -0.2 | -0.1 |
| Total cholesterol | -0.0001 | -0.0001 | -5.00E-05 | 9.00E-06 | -0.1 | -0.1 | -0.05 |
| Triglycerides | -3.00E-05 | -8.00E-05 | 2.00E-05 | 0.22 | -0.03 | -0.08 | 0.02 |
| Disease status |  |  |  |  |  |  |  |
| Viral hepatitis | -0.0002 | -0.001 | 0.001 | 0.78 | -0.2 | -1 | 1 |
|  |  |  |  |  |  |  |  |
| Type 1 Diabetes | -0.001 | -0.002 | -0.0005 | 0.002 | -1 | -2 | -0.5 |
| Type 2 Diabetes | -0.002 | -0.002 | -0.002 | 5.93E-39 | -2 | -2 | -2 |
| Vitamins/Minerals |  |  |  |  |  |  |  |
| Iron supplement | 0.001 | 0.0006 | 0.002 | 0.0003 | 1 | 0.6 | 2 |
| Vitamin C | 0.0001 | -0.0002 | 0.0005 | 0.53 | 0.1 | -0.25 | 0.5 |
| Medication |  |  |  |  |  |  |  |
| Proton pump inhibitors | -0.0009 | -0.001 | -0.0007 | 3.19E-33 | -0.9 | -1 | -0.7 |
| Diet |  |  |  |  |  |  |  |
| Alcohol intake |  |  |  |  |  |  |  |
| 0 units/week | -0.0005 | -0.0007 | -0.0004 | 7.98E-12 | -0.523 | -0.67 | -0.4 |
| 1-14 units/week | Reference | | | | | | |
| 15-29 units/week | 0.001 | 0.0009 | 0.001 | 3.94E-68 | 0.989 | 0.88 | 1 |
| ≥30 units/week | 0.002 | 0.0019 | 0.002 | 1.09E-146 | 2.03 | 1.88 | 2 |
| Red/processed meat consumption |  |  |  |  |  |  |  |
| 0 times/week | Reference | | | | | | |
| 0.1-2.9 times/week | 0.0003 | 7.88E-05 | 0.0004 | 0.004 | 0.3 | 0.08 | 0.4 |
| ≥3.0 times/week | 0.0009 | 0.0007 | 0.001 | 4.83E-22 | 0.9 | 0.9 | 1 |
| Tea intake per day |  |  |  |  |  |  |  |
| 0 cups/day | Reference | | | | | | |
| <1 cup/day | 0.0002 | -0.0001 | 0.0005 | 0.21 | 0.184 | -0.1 | 0.5 |
| 1-3 cups/day | -0.0005 | -0.0006 | -0.003 | 3.76E-11 | -0.46 | -0.6 | -3 |
| ≥4 cups/day | -0.004 | -0.0005 | -0.0002 | 5.61E-08 | -3.73 | -0.5 | -0.2 |
| Lifestyle |  |  |  |  |  |  |  |
| Current smoker | 7.00E-05 | -1.00E-05 | 0.0001 | 0.1 | 0.07 | -0.01 | 0.1 |

Linear regression beta-coefficients (β) and 95% confidence intervals (CI) for associations between environmental and genetic factors and MRI-derived liver iron accumulation rate (liver iron concentration divided by age). Models included European ancestry participants only and were adjusted for sex, and 10 genetic principal components (PCs) to reduce bias from population stratification.

**Abbreviations:** BMI, body mass index; CI, confidence interval; excl, excluding; HbA1c, glycated hemoglobin; MRLIC, magnetic resonance liver iron concentration; MRI, magnetic resonance imaging; mmol/L, millimoles per litre; PGS, polygenic score; p-value, probability value; SD, standard deviation; SF, serum ferritin; TSAT, transferrin saturation; WHR, waist-to-hip ratio; WHtR, waist-to-height ratio; β, beta-coefficient. PGS for transferrin saturation and serum ferritin were derived from a published GWAS.^4^ All results were multiplied by 1,000 to enhance interpretability due to small absolute effect sizes.

## **References**

1. UK Government. Guidance Chapter 12: Alcohol. GOV.UK. November 9, 2021. Accessed July 2, 2024. https://www.gov.uk/government/publications/delivering-better-oral-health-an-evidence-based-toolkit-for-prevention/chapter-12-alcohol

2. Li N, Zhao G, Wu W, et al. The Efficacy and Safety of Vitamin C for Iron Supplementation in Adult Patients With Iron Deficiency Anemia. *JAMA Netw Open*. 2020;3(11):e2023644. doi:10.1001/jamanetworkopen.2020.23644

3. Bradbury KE, Murphy N, Key TJ. Diet and colorectal cancer in UK Biobank: a prospective study. *Int J Epidemiol*. 2020;49(1):246-258. doi:10.1093/ije/dyz064

4. Moksnes MR, Graham SE, Wu KH, et al. Genome-wide meta-analysis of iron status biomarkers and the effect of iron on all-cause mortality in HUNT. *Commun Biol*. 2022;5(1):591. doi:10.1038/s42003-022-03529-z
